# Revealing Epigenetic Factors of circRNA Expression by Machine Learning in Various Cellular Contexts

**DOI:** 10.1016/j.isci.2020.101842

**Published:** 2020-11-24

**Authors:** Mengying Zhang, Kang Xu, Limei Fu, Qi Wang, Zhenghong Chang, Haozhe Zou, Yan Zhang, Yongsheng Li

**Affiliations:** 1 College of Bioinformatics Science and Technology, Harbin Medical University, Harbin 150081, China; 2 Key Laboratory of Tropical Translational Medicine of Ministry of Education, Hainan Medical University, Haikou 571199, China; 3 School of Life Science and Technology, Harbin Institute of Technology, Harbin 150001, China

**Keywords:** Bioinformatics, Omics, Transcriptomics

## Abstract

Circular RNAs (circRNAs) have been identified as naturally occurring RNAs that are highly represented in the eukaryotic transcriptome. Although a large number of circRNAs have been reported, the underlying regulatory mechanism of circRNAs biogenesis remains largely unknown. Here, we integrated in-depth multi-omics data including epigenome, transcriptome, and non-coding RNA and identified candidate circRNAs in six cellular contexts. Next, circRNAs were divided into two classes (high versus low) with different expression levels. Machine learning models were constructed that predicted circRNA expression levels based on 11 different histone modifications and host gene expression. We found that the models achieve great accuracy in predicting high versus low expressed circRNAs. Furthermore, the expression levels of host genes of circRNAs, H3k36me3, H3k79me2, and H4k20me1 contributed greatly to the classification models in six cellular contexts. In summary, all these results suggest that epigenetic modifications, particularly histone modifications, can effectively predict expression levels of circRNAs.

## Highlights

• CircRNAs exhibit specific expression in various cellular contexts
• High and low expressed circRNAs exhibit different biological functions
• Histone modifications are significantly correlated with circRNAs expression
• Machine learning models were constructed for predicting circRNAs expression

## Graphical Abstract

**Figure:**
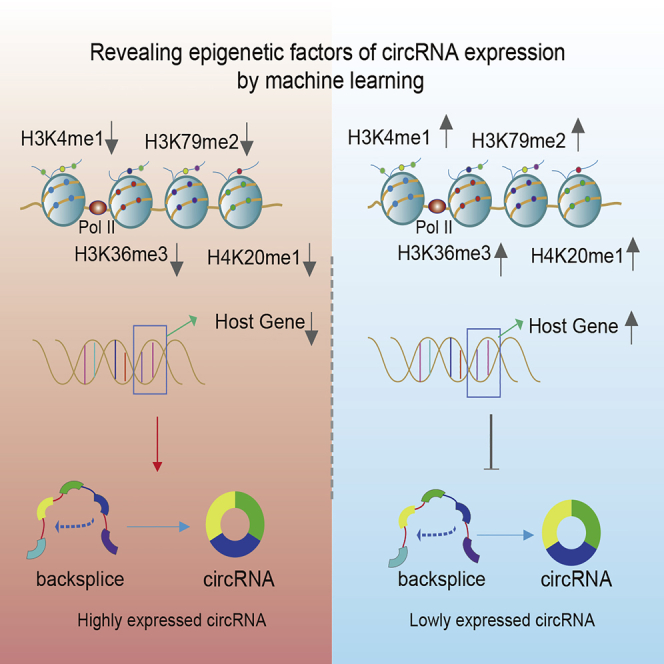

## Introduction

Circular RNA (circRNA) is a novel endogenous non-coding RNA that is common in the eukaryotic transcriptome (Glazar et al., 2014; Memczak et al., 2013; Salzman et al., 2013) and characterized by the presence of covalent bonds connecting the 3′ and 5′ ends (Jeck et al., 2013). Several circRNAs have been identified from a few transcriptional genes more than 30 years ago (Cocquerelle et al., 1993; Nigro et al., 1991; PG, 1996); the biogenesis is regulated by specific *cis*-acting elements and *trans-*acting factors (Kristensen et al., 2019). The cyclization of circRNAs is promoted by surrounding complementary sequences and regulated by specific RNA-binding proteins (Ashwal-Fluss et al., 2014; Conn et al., 2015; Ivanov et al., 2015; Liang and Wilusz, 2014; Zhang et al., 2014). In addition, both alternative splicing events within the same back-splice junction and alternative back-splice site selection can produce various circRNAs from the same gene locus (Gao et al., 2016). In the past few years, various studies have demonstrated that circRNAs are ubiquitous in animals and not the previously considered splicing by-product (Salzman et al., 2012).

Accumulating studies have shown that circRNAs play important roles in carcinogenesis and are expected to be therapeutic targets (Chen et al., 2019a; Han et al., 2018; Huang et al., 2020; Ju et al., 2019; Miranda et al., 2006; Qian et al., 2018). For example, a circRNA, CDR1 antisense RNA (CDR1as) (antisense to the cerebellar degeneration-related protein 1 transcript), was reported as miR-7 sponge and inhibited the function of miR-7 in colorectal cancer (Weng et al., 2017). *circMLL/AF9* that was derived from oncogenic fusion genes can contribute to tumor-promoting properties (Guarnerio et al., 2016). Moreover, the expression of circRNAs was extensively dysregulated in complex diseases. Cell cycle-related *circTP63* was up-regulated in lung squamous cell carcinoma (LUSC) tissues, and its up-regulation was directly correlated with larger tumor size and higher tumor node metastasis (TNM) stage in patients with LUSC (Cheng et al., 2019). In addition, *circ*ASAP1, a circRNA derived from exons 2 and 3 of the ASAP1 gene, was overexpressed in hepatocellular carcinoma (HCC) cell lines with high metastatic potential and in metastatic HCCs (Hu et al., 2019).

Recent studies have shown that RNA-binding protein (RBP) is a key regulator of the expression pattern of circRNAs. Such as QKI (Conn et al., 2015), and DExH-box helicase 9 (DHX9) (Aktas et al., 2017), they have been accumulatively reported to regulate the formation of circRNAs and are key players in post-transcriptional events (Pereira et al., 2017). In addition, efforts in *D. melanogaster* suggest that the biogenesis of many circRNAs is influenced by a combination of *cis*-acting elements and *trans-*acting splicing factors, including heterogeneous nuclear ribonucleoproteins (hnRNPs) and SR proteins (that is, proteins containing a long repeat of serine and arginine amino acid residues) (Kramer et al., 2015). Furthermore, circRNAs may be specifically generated and regulated from a study using (estrogen-stimulated) MCF-7 cells, which show higher levels of H3K36me3 and a higher number of Ago-binding sites in circularizing exons (Tarrero et al., 2018).

Recent studies have shown that histone modifications affect the splicing mechanisms and splicing outputs by recruiting splicing regulators that affect chromatin-binding proteins (Luco et al., 2010). However, little is known about whether there exists specific regulatory mechanism between histone modification and circRNA expression. To fill this gap, we systematically analyzed global circRNAs expression in a large panel of cell lines from the Encyclopedia of DNA Elements (Consortium, 2004) (ENCODE). The origins and distribution of circRNAs on the chromosome were identified and analyzed in various cellular contexts. Next, epigenetic modification signals, mainly histone modifications, and expression of circRNA-related host genes were used to characterize high or low expression levels of circRNAs. We identified five important factors that can markedly distinguish the expression of circRNAs. Furthermore, we explored the relationship of epigenetic factors and circRNA expression in each cell line. In summary, we initially investigated the regulation relationship between circRNA expression and histone modifications, which provides an important reference for further study of circRNA biogenesis.

## Results

### Identification of circRNAs in Various Cellular Contexts

We analyzed circRNA transcripts using RNA sequencing (RNA-seq) of ribosomal RNA-deplete RNA from six cell lines (A549, GM12878, H1-hEsc, HepG2, HeLa-S3, and NHEK). A detailed summary for the samples used in this study was provided in Table S1 and Table S2. The pipeline based on BWA-MEM alignment was used to identify circRNAs with gene annotations (Figure S1). In total, we identified 23,989 unique candidate circRNAs in all six cell lines. The numbers of circRNA identified in each cell line was provided in Table 1. Compared with previously identified circRNAs that were downloaded from circBase (Glazar et al., 2014), we found that there are 14,499 known circRNAs and 9,490 circRNAs identified in our current study (Table S3). Moreover, compared with circRNAs identified in circAtlas (Wu et al., 2020) and circRIC (Ruan et al., 2019), we also found that the majority of circRNAs has been identified in these databases (Table S4). Thus, all these results suggested that circRNA is the RNA family that plays an important role, not a by-product of splicing. Notably, the differences in sequencing depth, variable identification methods, and differences in the developmental stage of cells or tissues may all contribute to the final discovery of novel circRNAs.

**Table 1:** Number of CircRNAs across Six Cell Lines

Cell Line	Number of circRNA	Number of Highly Expressed circRNA	Number of Lowly Expressed circRNA
A549	7,972	510	597
GM12878	10,767	657	662
H1-hESC	7,782	427	441
HepG2	8,278	319	322
Hela-S3	8,471	330	331
NHEK	3,131	96	97
Total	23,989	1,276	1,903

**Figure 1:**
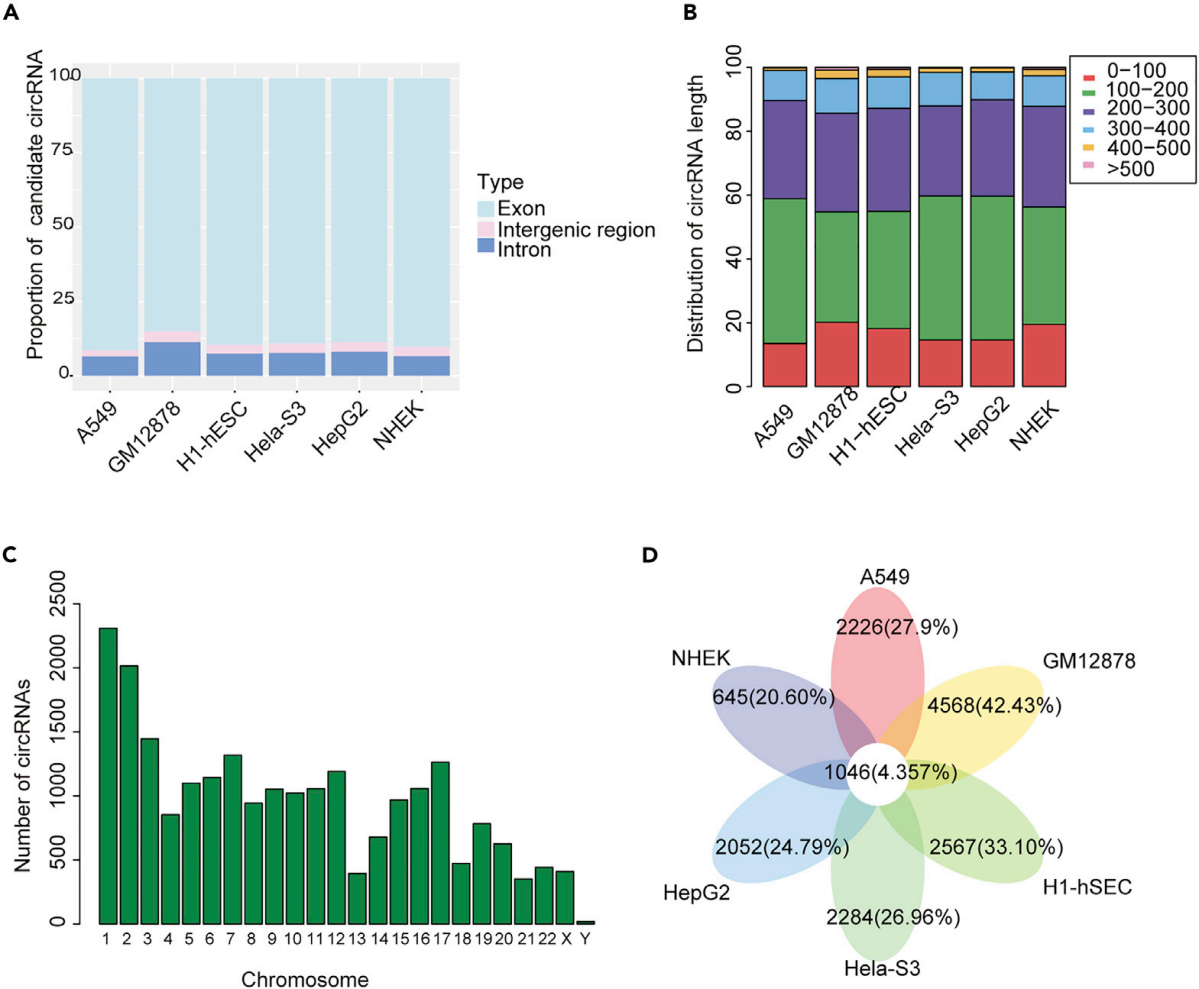
Profiling of Circular RNAs in Different Cell Lines (A) Barplot showing the proportion of circRNAs with variant origins. Light blue for exon circRNAs, pink for intergenic circRNAs, and blue for the intron circRNAs. (B) The splice length distribution of circRNAs in six cell lines. (C) Bar graph showing the distribution of circRNA on different chromosomes. (D) Venn diagram showing candidate circRNAs across six cell lines. See also Figures S1 and S2 and Tables S1 and S2, Tables S3 and S4.

Next, we annotated these circRNA candidates using the RefSeq database (Pruitt et al., 2012). Previous work has revealed the variant types of circRNAs in genomic regions (Memczak et al., 2013; Zheng et al., 2016). We found that more than 85% of circRNA candidates were derived from exon, whereas smaller fractions aligned with introns, intergenic region (Figure 1A). We analyzed the length of exon circRNAs and found that the length in most of the six cell lines was less than 500 bp (Figure 1B). We also reconstructed the full-length sequences of circRNAs by CIRI-full (Zheng et al., 2019). We found that the average length distribution of circRNA was similar, and the majority of circRNAs was around 200 bp in length (Figure S2). Moreover, no significant correlation was observed between the distribution of candidate circRNAs and chromosome length and number of genes (Figure 1C). Next, we analyzed the number of circRNAs shared by six cell lines. Interestingly, we rarely found that the number of common circRNAs in six cell lines, which accounted for approximately 4.36% of all circRNAs. However, in a single cell line, such as GM12878, cell-type-specific circRNAs accounted for 42.43% of the total number (Figure 1D and Table S5). These findings were consistent with previous studies that a vast majority of circRNAs show tissue and cell type specificity (Salzman et al., 2013; Szabo et al., 2016). In addition, we focused on the differences between shared and specific circRNAs, including the expression status and the type of circRNAs. We found that shared circRNAs had a higher proportion of exon circRNAs and significantly higher length and expression than specific circRNAs (Figure S3).

### Quantitative Analysis of circRNA Expression

**Figure 2:**
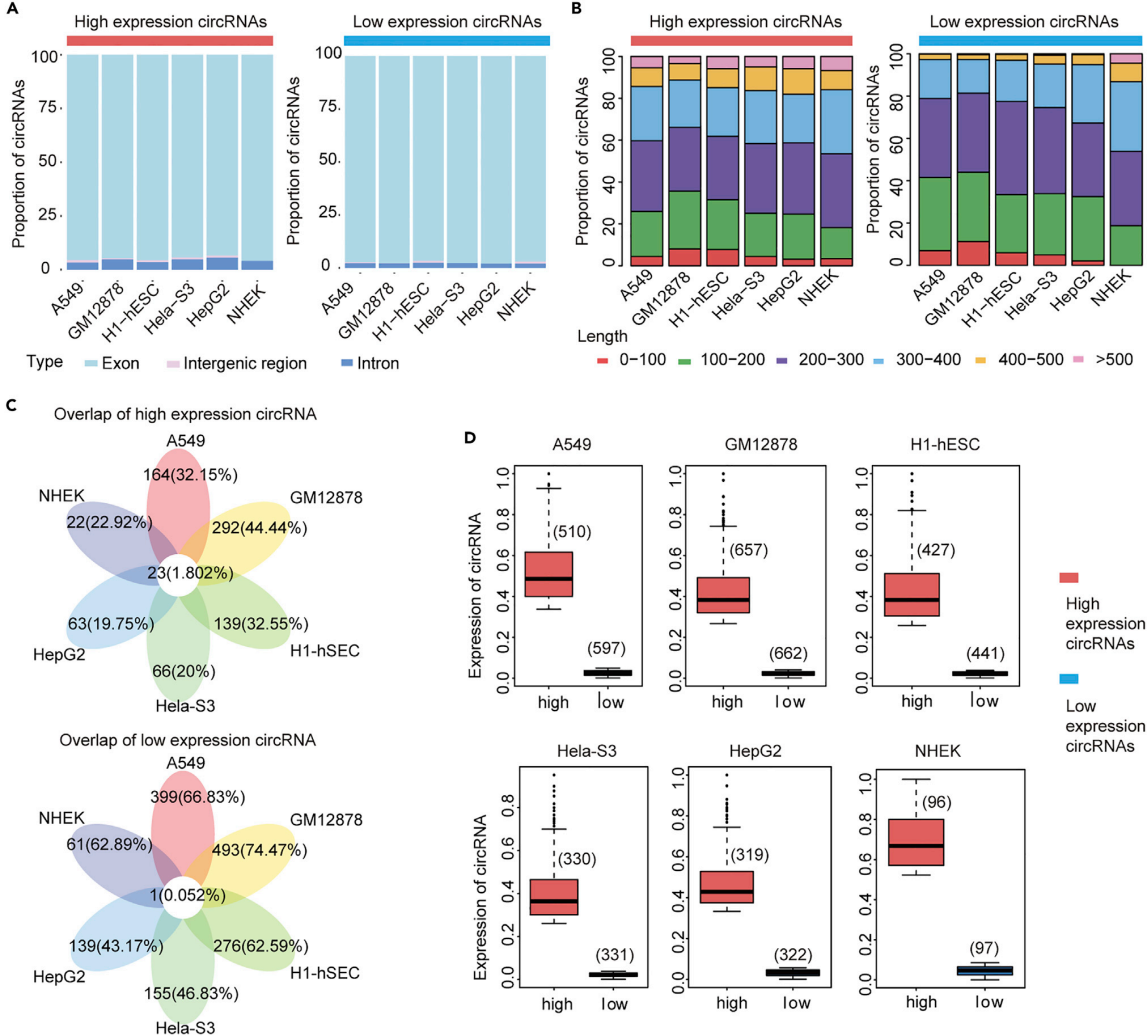
Profiling of High and Low Expression circRNAs in Six Cell Lines (A) A bar graph showing the proportion of different types of high- and low-expression circRNAs. The left part is highly expressed circRNA, and the right part is low expression circRNA. (B) The splice length distribution of circRNAs with different expression pattern in six cell lines. The left part is a high expression circRNA, and the right part is a low expression circRNA. (C) The Venn diagram of high and low expression circRNA intersection in six cell lines. (D) Box plot showing expression value and number of high and low expression circRNAs. See also Figures S3–S5 and Table S5.

Next, we examined the expression abundance of circRNAs in cell lines. We first divided circRNAs into two classes with different expression patterns, according to the expression values. We defined circRNAs with top 20% expression value as high expression circRNAs, the bottom 20% expression value as low expression circRNAs (Figure S4). The numbers of circRNA with different expression patterns identified in the six cell lines were listed in Table 1. We next explored the differences in expression patterns of circRNAs across six cell lines. Consistent with previous studies, exon circRNAs were the main type of circRNAs (Figure 2A). The length of most exon-circRNAs was <500 bp, and the median length was about 200 bp (Figure 2B). Moreover, the number of shared circRNAs across cell lines was quite small. This result revealed that numerous circRNAs seem to be specifically expressed across various cells. Notably, the circRNAs shared by cell lines in the low expression pattern was less than those in high expression pattern. This indicates that expression specificity of circRNA is higher in the low expression pattern (Figure 2C). A box plot of circRNA expression was shown in Figure 2D.

Although studies have shown that splice length of circRNA in the genome is relatively short, the number and length of exon-forming circRNAs are obviously different. Next, we explored whether the expression of circRNA is affected by the genome length of circRNA. Spearman correlation was performed for length and expression of all identified circRNAs. The analysis revealed that there was no significant correlation between length and expression of circRNA (Figure S5). This result indicated that expression of circRNAs is not actually affected by length. The expression levels of circRNA in cells varied greatly, suggesting that they play different functional roles in different cellular contexts.

**Figure 3:**
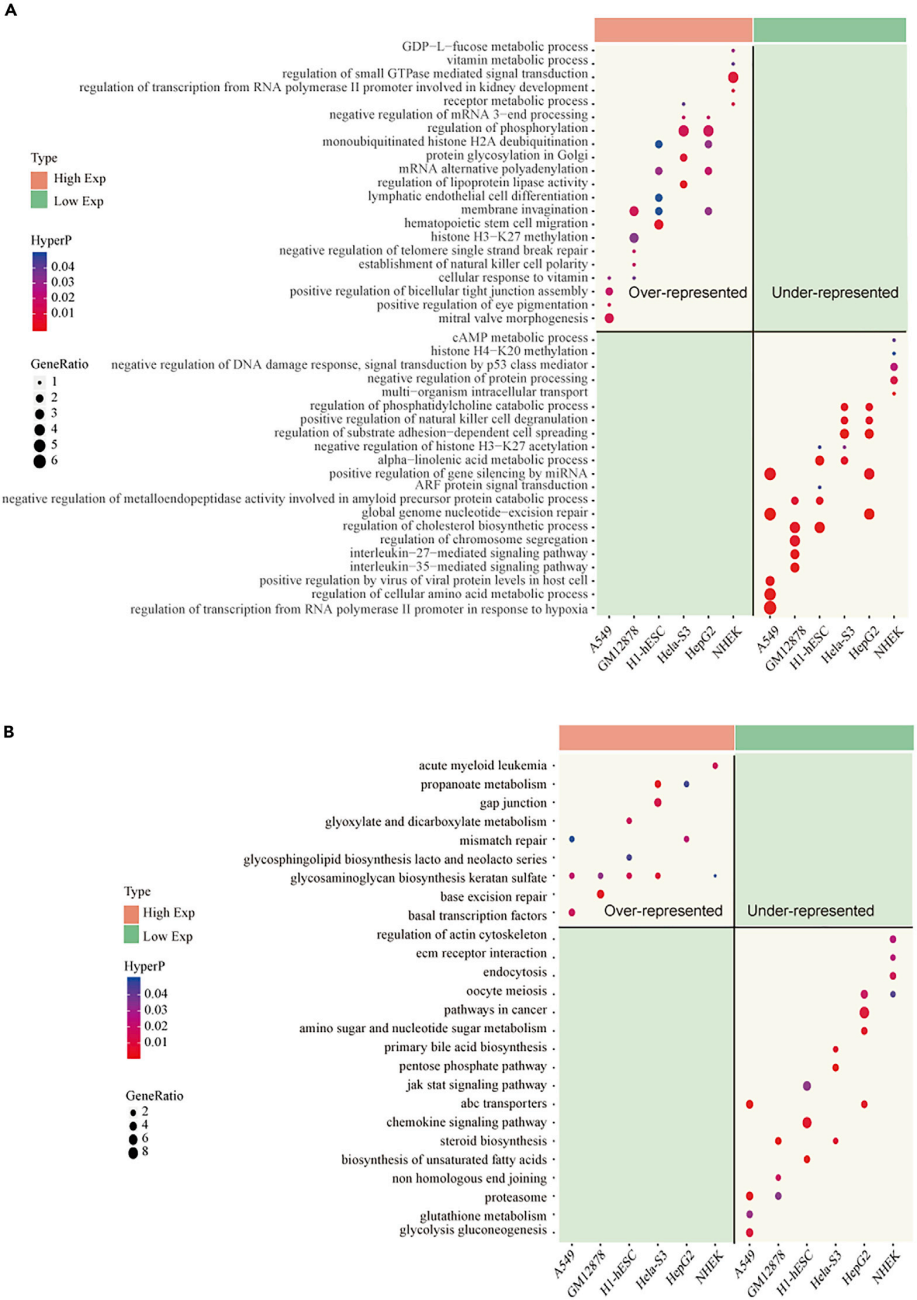
Over-Represented and Under-Represented Functions of circRNA with High and Low Expression Classes (A) Enriched bubble diagrams of high and low expression patterns in biological processes. The portion covered by the red band represents a high expression circRNA, and the portion covered by the green band represents a low expression circRNA. The color of the bubble represents the p value; bubble size represents the number of circRNA host genes that present in one term. (B) Bubble diagrams of high and low expression patterns in the KEGG pathway. See also Figures S6 and S7.

Next, Gene Ontology (GO) function and Kyoto Encyclopedia of Genes and Genomes (KEGG) pathway enrichment analysis were performed to infer potential functions of circRNA. We identified terms that were over-represented or under-represented in the two expression patterns. Our analysis found that high expression circRNAs tend to be enriched in biological processes, such as receptor's metabolic process, negative regulation of mRNA 3-end processing, histone H3-K27 methylation, regulation of small GTPase-mediated signal transduction, and cell differentiation (Figure 3A). Low expression circRNAs were functionally enriched in positive regulation of gene silencing by miRNA, negative regulation of histone H3-K27 acetylation, histone H4-K20 methylation, cAMP metabolic process, and regulation of substrate adhesion-dependent cell spreading. Moreover, KEGG pathway results suggested that high expression circRNAs may be involved in mismatch repair pathway and basal transcription factors (Figure 3B). Notably, circRNAs in low expression groups were enriched in pathways in cancer, which was consistent with the previous conclusion that circRNAs in cancers tend to be low expression (Vo et al., 2019). Molecular function and cell components enrichment also showed differences between circRNAs in two groups (Figures S6 and S7). Taken together, these results suggest that there may be some relationship between the expression patterns of circRNA and histone modifications. It also shows that two different patterns of circRNA perform different biological functions in the life of an organism.

### Epigenetic Features Are Predictive of circRNA Expression across Cellular Contexts

As an important epigenetic regulator, histone modification has been widely studied in recent years. Emerging evidence has shown important roles of histone modifications in circRNA biogenesis (Tarrero et al., 2018). To further explore the relationship between circRNA expression and histone modifications, we downloaded histone modification signals shared by six cell lines from ENCODE project. The host gene expression was obtained from RNA-seq using Cufflinks (Trapnell et al., 2010) (see Transparent Methods). We hypothesized that the binding peaks of histone modification obtained from ENCODE would be different between high expression circRNAs and low expression circRNAs. To test this hypothesis, we applied 11 histone modification peaks and expression of host gene to characterize circRNA with different expression patterns. For the robustness of classification model, the datasets were divided into training sets and testing sets. The models trained in training sets were applied to the testing sets to evaluate the robustness.

It is well known that the A549 cell line is human lung adenocarcinoma cell line. In recent years, circRNA expression in A549 has been extensively studied (Dai et al., 2018; Sun et al., 2019). We first constructed five frequently used machine learning classification algorithms (decision tree, logistic regression, SVM, naive Bayes, and random forest) to predict circRNA expression levels in A549 cell line. Indeed, those algorithms have different classification power on circRNA expression prediction. We evaluated the power of the classification models combining with three indicators, including the precision, accuracy, and area under the ROC curve (AUC) of the classifier (Figure S8). All these results suggest that classification model of random forest was the best one. Therefore, random forest was used to integrate the histone modification peaks and host gene expression for circRNA prediction.

**Figure 4:**
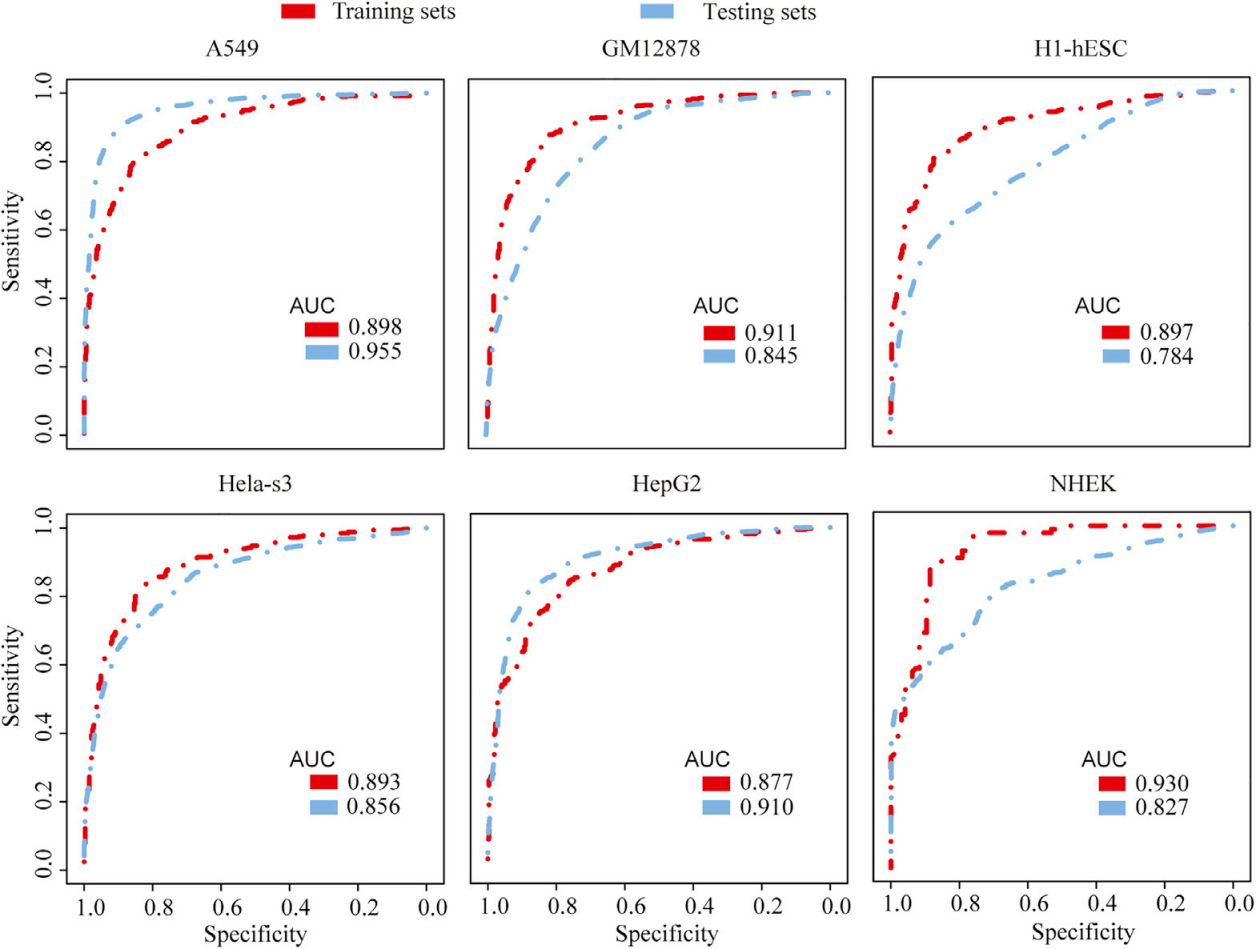
The Area under the ROC Curve of Random Forest Classification for Each Cell Line The predictive ability of 12 factors to characterize the different expression patterns of circRNA. The red line represents the AUC value in the training sets, and the blue line represents the AUC value in the testing sets. See also Figure S8.

Next, random forest was applied to train the model in six cell lines. First, our datasets were divided into training sets and testing sets (see Transparent Methods). We performed 10-fold cross-validation on our datasets to verify that the correlation was not specific to a subset of data. Finally, the AUC indicated that both the training sets and the testing sets model are robust in the six cell lines (Figure 4). For example, in A549 cell line, the AUC reached 0.89 in the training sets, whereas the AUC of the model classification was as high as 0.95 in the testing sets. In the GM12878 cell line, the area under the ROC curve was 0.911 in the training sets, and this value reached 0.845 in the testing sets. The accuracy of the model in the training sets of the H1-hESC cell line was 0.897 and it was 0.78 in testing sets. Similarly, in the Hela-s3, HepG2, and NHEK cell-lines, the AUC predicted by the training sets and the testing sets model all reached 0.8 or more. All these results demonstrated that histone modifications were indicative of genomic circRNA expression in different cell types.

### Epigenetic Determinants of circRNA Expression

**Figure 5:**
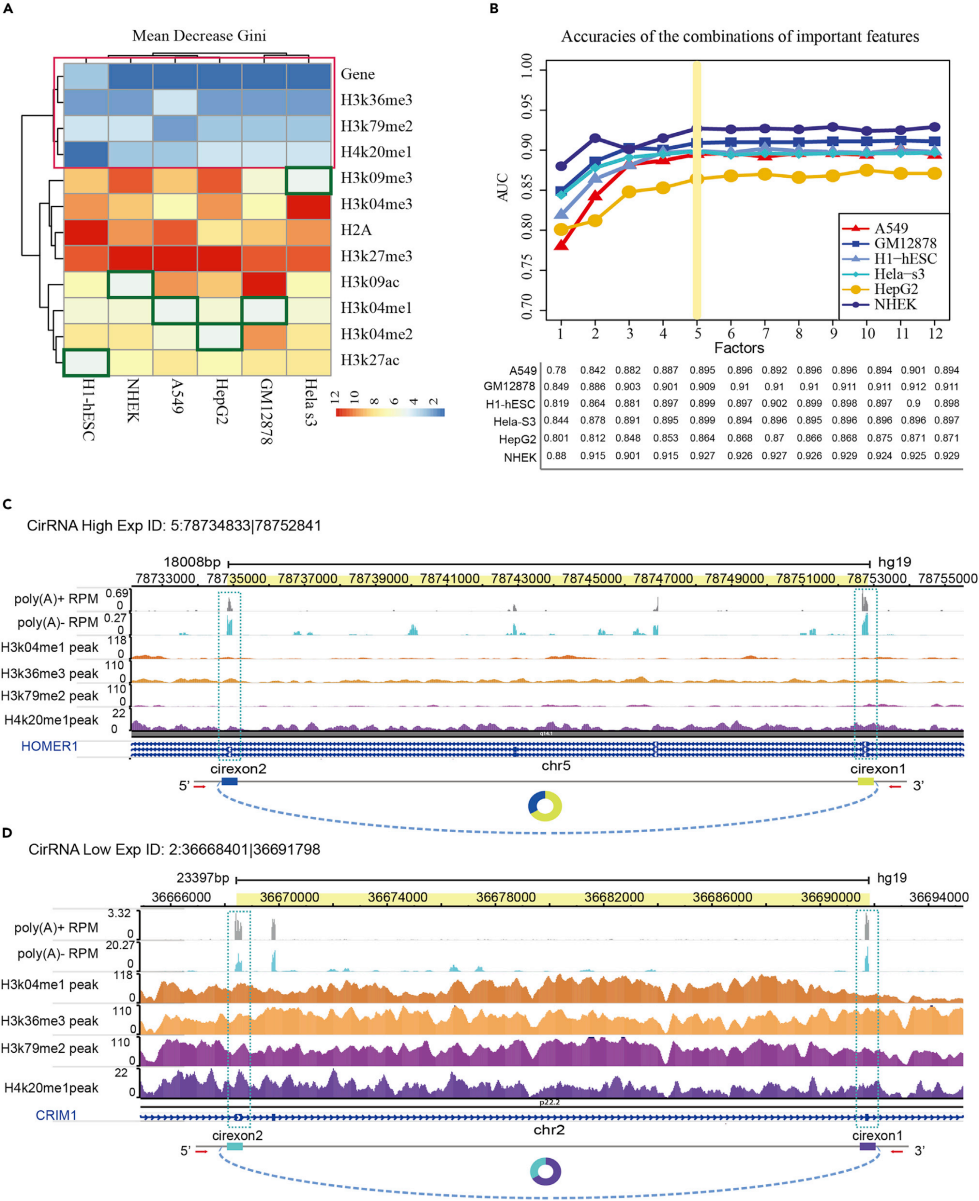
Model Evaluation Capabilities of Five Classifiers and Contribution of Factors to the Model (A) MDG of each factor, the color from blue to red represents the importance of high to low, the column represents six cell lines, and the row represents 12 factors. The red box factors are SPE of each cell line, and the green box represents the fifth most important factor. (B) Model predictive ability assessment of top n factors combination, the yellow line marks the fifth contribution factor, and the area under the ROC curve of the factor has stabilized. (C and D) The circRNA visualization of four key histone modification signals, the origin of exons, their expression levels from the poly(A) RNA-seq (blue wiggle tracks), and the expression of their cognate mRNAs from the poly(A)+ RNA-seq (gray wiggle tracks) in A549 cells. See also Figure S9.

The mean decrease Gini (MDG) was used to evaluate the importance of each feature to provide a relative ranking of the investigated features. The larger MDG indicates the increasing importance of the corresponding feature for prediction of circRNA expression patterns. Several important factors were revealed according to MDG in the random forest classifier (Figure 5A). The colors changed from blue to red and represented the decreasing importance of the factor. The host genes' expression, H3K79me2, H3K36me3, and H4K20me1, all contributed greatly in the classification of each cell line; these four factors showed a higher MDG and were identified as shared potential effectors (SPEs) (the factor in the red box in Figure 5A). Next, to avoid factor redundancy events in the model construction, we selected the top n (n = 1, 2, 3, 4 … 12) important factors in each cell line to construct the classifier to evaluate the prediction ability of the model (Figure 5B). Although the AUC varied in each cell line, when the factors of the constructed model reached five, the AUC value of the model tended to be stable. Therefore, we identified five important factors in each cell line (red box in Figure 5A and the factor represented by the green box) as maker factors for different patterns of circRNA expression.

Moreover, we confirmed our result in another independent validation sets (see Transparent Methods). We collected the available RNA-seq datasets from the Gene Expression Omnibus database (GEO) (Edgar et al., 2002) and downloaded the peaks of 11 histone modifications from The NIH Roadmap Epigenomics Mapping Consortium (http://www.roadmapepigenomics.org/). We obtained a total of five public data resources of each cell line (A549, GM12878, H1-hESC, HeLa-S3, HepG2). We next selected top five factors in each cell line for model construction. The results showed that the AUC was above 0.85 (Figure S9). These results further validated the reliability of our models and also showed that the top five factors may affect the expression pattern of circRNAs.

To investigate the differences in the modification signal of the five factors in circRNA, we selected high and low expression circRNA in A549 cells related to lung cancer in the circBase database. For example, high expression circRNA *5:78734833|78752841* and low expression circRNA *2:36668401|36691798* (Figures 5C and 5D). circRNA *5:78734833|78752841* is a known *circ0006916*, which is derived from the homer scaffold protein 1 (HOMER1) gene and is highly expressed in the A549 cell line (Dai et al., 2018). The circRNA *2:36668401|36691798* is known as *circ0007386*. These two circRNAs are formed by back splice of two exons on chromosome 5 and chromosome 2. Visualization shows that poly(A)+ RNA expression is higher in low expression pattern. These results demonstrated that the expression of circRNA and linear isoforms were independent of each other. The variations of H3K4me1, H3K36me3, H3K79me2, and H4K20me1 signals were strongly correlated with the circRNA expression dynamics. The signal of histone modification tended to be at a lower level in highly expressed circular RNA.

### Modeling the circRNA Expression Based on Epigenetic Features

**Figure 6:**
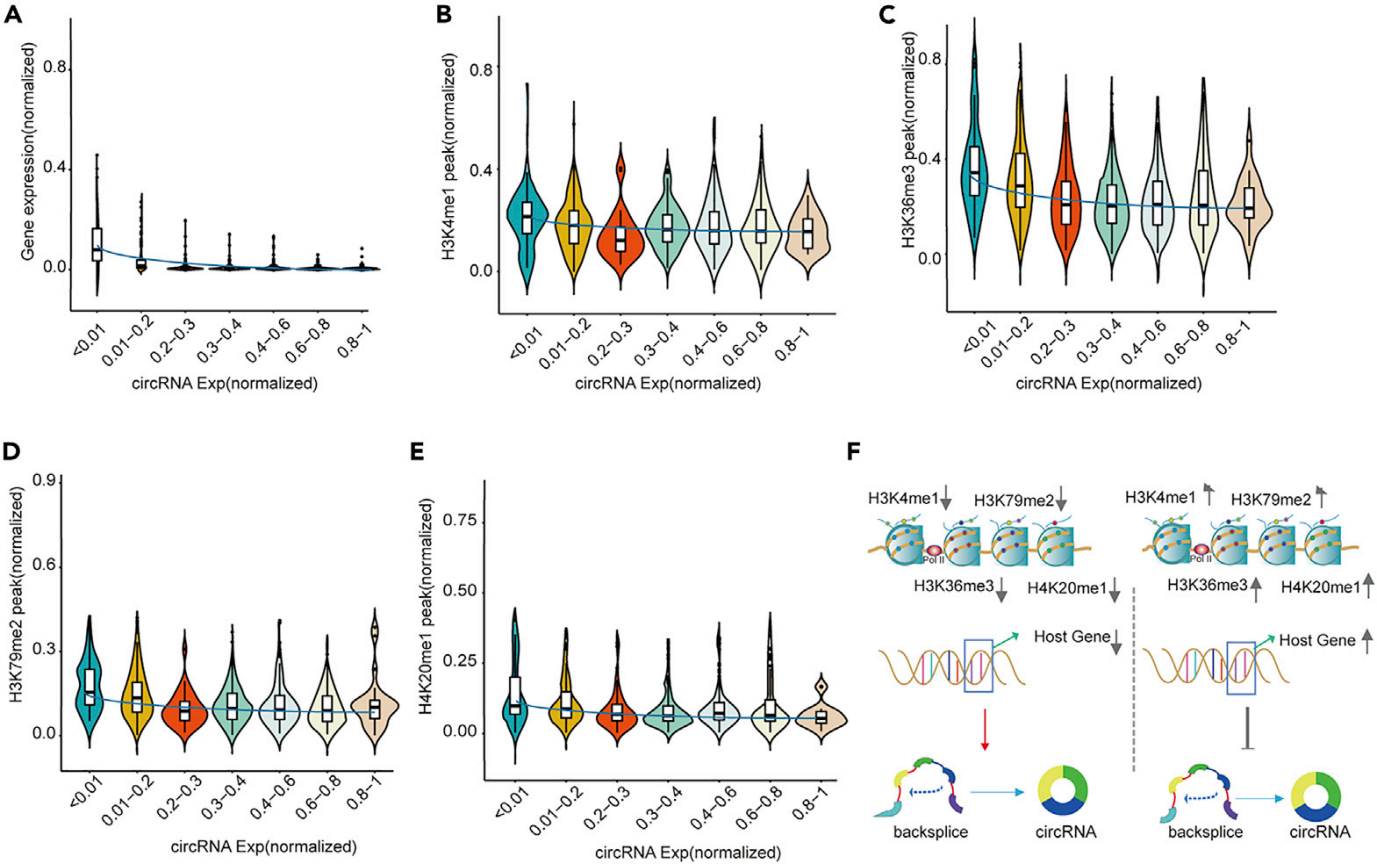
The Relationship between circRNA Expression and Five Important Signals in the A549 Cell Line (A) Violin plot of host gene and circRNA expression. (B) Violin plot of histone-modified H3K4me1 and circRNA expression. (C) Violin plot of histone modification of H3K36me3 and circRNA expression. (D) Violin plot of histone modification of H3K79me2 and circRNA expression. (E) Violin plot of histone-modified H4K20me1 and circRNA expression. (F) Schematic diagram of the relationship between five signals and circRNA in the A549 cell line. The data used in the figure was normalized. See also Figures S10–S15.

We used random forest to screen five important factors based on MDG to characterize circRNA. Four of the important factors are common to each cell line. We next explored the existing connection of important factors and circRNA expressions. We subdivided the expression of circRNA and observed the trends of the five signal factors at different expression levels (Figures 6A–6E). In the A549 cell line, all five factors showed negative correlation with circRNA expression, indicating that the expression of host gene and four epigenetic factors has a negative regulatory relationship to circRNA expression (Figure 6). The host gene, H3k36me3, H3k79me2, H4k20me1 in the GM12878 cell line showed the negative correlation with circRNA expression, whereas H3K4me1 peak and circRNA expression revealed weak promotion (Figure S10). In the H1-hESC cell line, host gene, H3k36me3, H3k79me2, H4k20me1, H3K4me1, and circRNA expression showed a negative correlation trend (Figure S11). In the Hela-s3 cell line, the expression of five factors with circRNA was consistent with H1-hESC, and both factors were negatively regulated (Figure S12). The relationship between each factor with the expression of circRNA in HepG2 cell line was similar to that of GM12878 cell line. The expression of host gene, H3k36me3, H3k79me2, H4k20me1, and circRNA showed a negative correlation, whereas the expression of the fifth important factor H3k04me2 in HepG2 cell line displayed positive promotion with circRNA expression (Figure S13). In the NHEK cell line, the top four shared factors, including host genes expression, H3k36me3, H3k79me2, and H4k20me1, were negatively correlated with circRNA expression, whereas H3k9ac and circRNA expression show a positive promotion (Figure S14).

In total, we identified four important factors shared by the cell lines, including host gene expression, H3k36me3, H3k79me2, and H4k20me1, were negatively correlated with the expression of circRNA (Figure 6F). We speculated that the expression of circRNA may be negatively affected by these four factors. The fifth important factor was the cell-specific factor, and the expression of circRNA showed an inconsistent trend. It suggested that this feature may have a cell line-specific relationship with circRNAs.

## Discussion

In this study, we identified and analyzed the global landscape of circRNAs in six different cell lines and found large and different genomic locations of circRNAs that are specifically expressed in tissues or cell lines as in previous studies (Guo et al., 2014; Salzman et al., 2013). To measure the expression of circRNA, we used the junction ratio as measure of the relative expression value of circRNA by CIRI (Gao et al., 2015). Consistently, we found that most of the identified circRNA expressions were of less abundance. This may be one of the reasons why circRNA has long been considered a by-product of the pre-mRNA splice process (Li et al., 2017). Importantly, to ensure the accuracy of analysis, we also performed CIRIquant (Zhang et al., 2020) for circRNA quantification and analyzed the Spearman correlation between the junction ratio in CIRI and CIRIquant. Compared with CIRI, the junction ratio in CIRIquant was higher, but overall, there was strong correlation between the two methods (Figure S15).

The current study found that the formation of circular RNA is regulated by a number of factors, of which Lariat-driven circularization and intron-pairing-driven circularization are the two most typical models (Chen and Yang, 2015). Moreover, it was discovered that the inverted repeat Alu (IRAlu) plays an important role in exon splicing and formation of circular RNA (Liang and Wilusz, 2014; Petkovic and Müller, 2015; Zhang et al., 2014). In addition, RNA-binding proteins (RBPs) also regulate the formation of circular RNA (Conn et al., 2015; Ruan et al., 2019). A novel computational method called iCircRBP-DHN was also proposed for discriminating circRNA-RBP bingeing sites (Yang et al., 2020). However, there are still many unknowns about the mechanism of the formation and function of circRNA. Although little research has been done on circRNA and epigenetics, there is increasing evidence that circRNA is associated with epigenetics. A recent study led to further explicit support for the possible specific production and regulation of circRNAs (Tarrero et al., 2018), which showed higher H3K36me3 levels (post-transcriptional histone modifications) in circularized exons.

In this work, we have quantified the relative contribution of host gene and histone modifications to circRNA expression regulation. We identified the important factors affecting the different expression patterns of circRNA by random forest. Moreover, the AUC of the random forest reached 0.78 or more, regardless of the training set or the testing set. These results showed that the factors that we identified show good effect on distinguishing the expression of circRNA. Next, we selected the factor features of the top five contributions to construct classifiers and estimate the area under the curve, thus eliminating the occurrence of factor redundancy in model construction. Although some histone modifications were redundant for predicting the overall circRNA expression, there might be different circRNA groups that are regulated by histone modifications in different ways. It should be noted that the redundancy only exists with regard to circRNA expression prediction. Essentially, distinct histone modification types play very different roles during transcriptional regulation (Xu et al., 2018). For example, both H3K4me3 and H3K36me3 act as marks for active genes; the former mainly occurs in the promoter regions facilitating the initiation of transcription, whereas the latter functions mainly in the transcribed regions involved in transcriptional elongation (Cheng and Gerstein, 2012).

Finally, we screened five important factors as features to characterize the different expression of circRNA. We observed that the four shared factors, host gene expression, H3k36me3, H3k79me2, and H4k20me1, showed negative effects on the expression of circRNA in each cell line, whereas the fifth important factor was cell lineage specificity. According to our analysis, the higher expression of the linear subtype of the host gene resulted in a decrease in the proportion of circulation of circRNA. Although several studies have found that circRNAs were generally poorly correlated with the expression of host genes, some circRNAs still show high correlation with host genes (Ruan et al., 2019). This is not a contradiction, as our conclusion is that host gene expressions contribute to the classification of high and low expressed circRNAs. We analyzed the contribution of each factor to the classifier using the Gini index. The mean decrease Gini (MDG) was used as the importance score of each feature to provide a relative rank of investigated features. The larger MDG indicates the increasing importance of the corresponding feature for predicting of circRNA expression patterns. In addition, we also verified the contribution of the top five factors to the accuracy of the model in the independent verification sets.

As we know, histone modification of H3k36me3 is related to the transcriptional region of the gene (Wagner and Carpenter, 2012; Zuo et al., 2018), and down-regulation of H3k36me3 and H3k79me2 expression promotes the back splice of exons. Histone modification of H4k20me1 is catalyzed by *PR-Set7* and is associated with the cell cycle (Beck et al., 2012) and down-regulated H4k20me1 promotes up-regulated expression of circRNA. In total, we explore the molecular mechanism of circRNA from the perspective of epigenetics. In addition, recent breakthrough researches have demonstrated that N6-methyladenosine (m6A) modification occurs in circRNAs and promotes protein translation through recruitment of the initiation factor *eIF4G2* and the m6A reader *YTHDF3* (Li et al., 2019; Paramasivam and Vijayashree Priyadharsini, 2020; Zhang et al., 2019). Chen et al. (2019b) reported that m6A modifications of human endogenous circRNAs exerted an important function of suppressing innate immune responses by inhibiting RIG-I activation. These may become a new direction for research in the field of circRNA and epigenetics.

In summary, we have shown that histone modification and host gene expression signals can predict circRNA expression in various cell lines. In addition, we selected a small number of modifications, which together can explain a large part of the difference in circRNA expression. The level of these modifications can be used to infer the expression of circRNA, thereby providing some information about the transcription process, which provides the possibility for many unknown mechanisms and functions of circRNA.

### Limitations of the Study

In this study, we provide a new perspective on the regulation of circRNA expression and use a machine learning method to model the high and low expression levels of circRNA and histone modification status to facilitate the understanding and discovery of new circRNA regulatory mechanisms. This work still has some limitations that deserve attention and further study. First, the histone modification data obtained from public databases for establishing regulatory models are not complete, although to our knowledge, these databases are already of high quality and relatively comprehensive. More complete data will be more conducive to comprehensive modeling of the relationship between biomolecules. For the types of histone modifications that do not exist in the model, we do not know whether there are regulatory relationships between histone modifications and circRNA. More complete data will be more conducive to the comprehensive modeling of regulatory relationships between biomolecules. Second, we selected a small number of modifications, which together can explain a large part of the difference in circRNA expression. Whether these modifications play a crucial role in the transcription process, or whether they represent equally important modification groups, must be clarified through further experimental studies.

### Resource Availability

#### Lead Contact

Further information and requests for resources should be directed to and will be fulfilled by the Lead Contact, Yongsheng Li (liyongsheng@hainmc.edu.cn).

#### Materials Availability

This study did not generate new materials.

#### Data and Code Availability

This study did not generate datasets/code.

## Methods

All methods can be found in the accompanying Transparent Methods supplemental file.

## Supplemental Information

Document S1. Figures S1–S15, and Tables S1 and S2, and Transparent Methods

Table S3. Summary of circRNA identified in six cell lines, related to Figure 1

Table S4. Overlap of circRNAs between six cell line and circRNA databases, related to Figure 1

Table S5. The list of specific and shared circRNAs in each cell line, related to Figure 1
