# Supplementary material for: Revealing Epigenetic Factors of circRNA Expression by Machine Learning in Various Cellular Contexts
Source: iScience. 2020 Nov 24;23(12):101842. doi: 10.1016/j.isci.2020.101842 (PMC7725743; doi:10.1016/j.isci.2020.101842)
Supplement: Document S1. Figures S1–S15, and Tables S1 and S2, and Transparent Methods [file mmc1.pdf]

iScience, Volume *23*

Supplemental Information

Revealing Epigenetic Factors of circRNA Expression by Machine Learning in Various Cellular Contexts

Mengying Zhang, Kang Xu, Limei Fu, Qi Wang, Zhenghong Chang, Haozhe Zou, Yan Zhang, and Yongsheng Li

Supplemental Figures

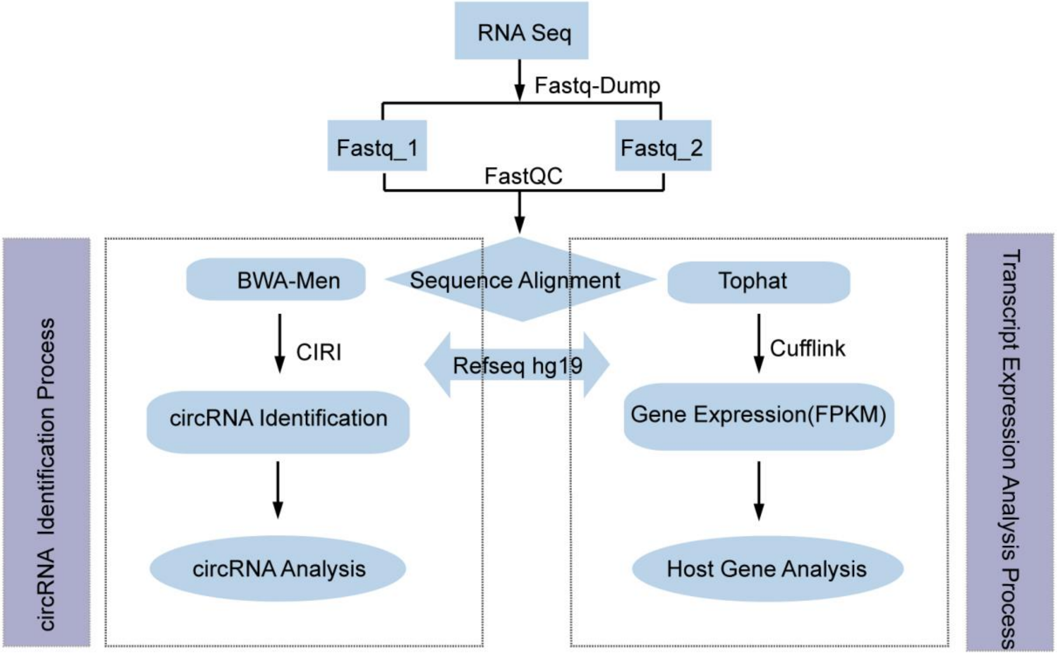

Figure S1. Flowchart for identification of circRNAs and host genes. Related to Figure 1.

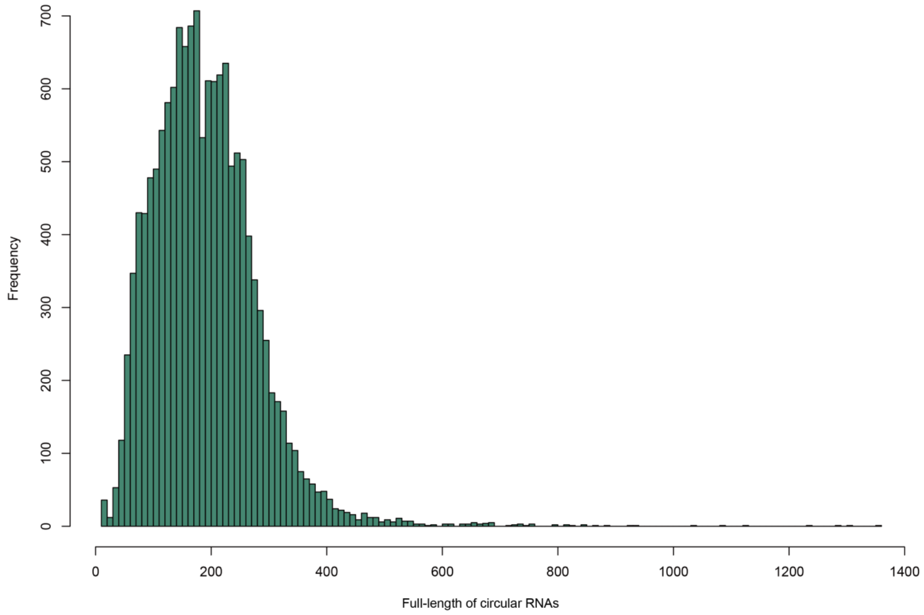

Figure S2. Density plot showing full length of circRNAs in six cell lines. Related to Figure 1.

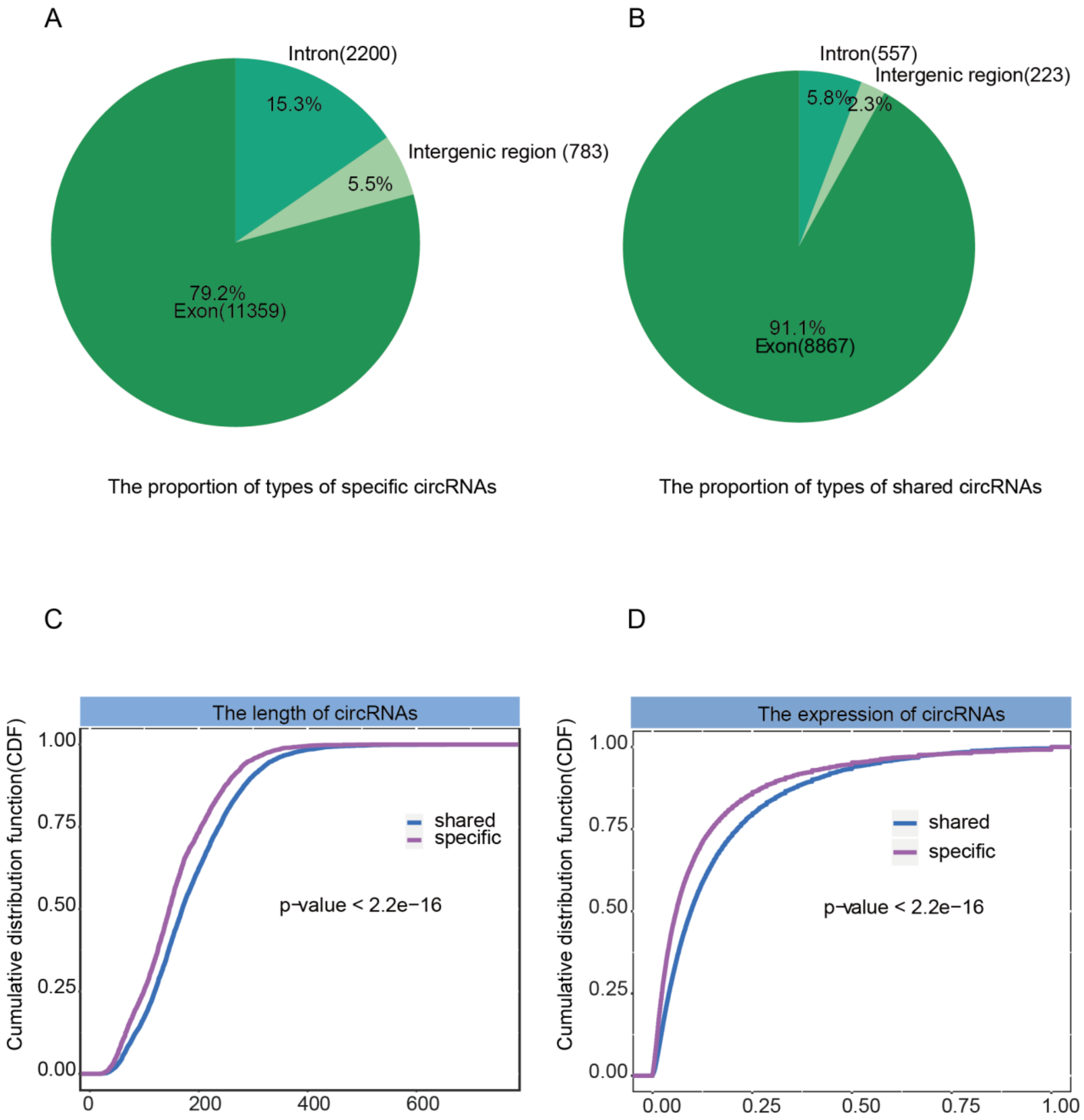

Figure S3. The differences between shared and specific circRNAs. (A-B) The proportion of types of specific and shared circRNAs. (C-D) The cumulative distribution of length and expression between specific and shared circRNAs. Related to Figure 2.

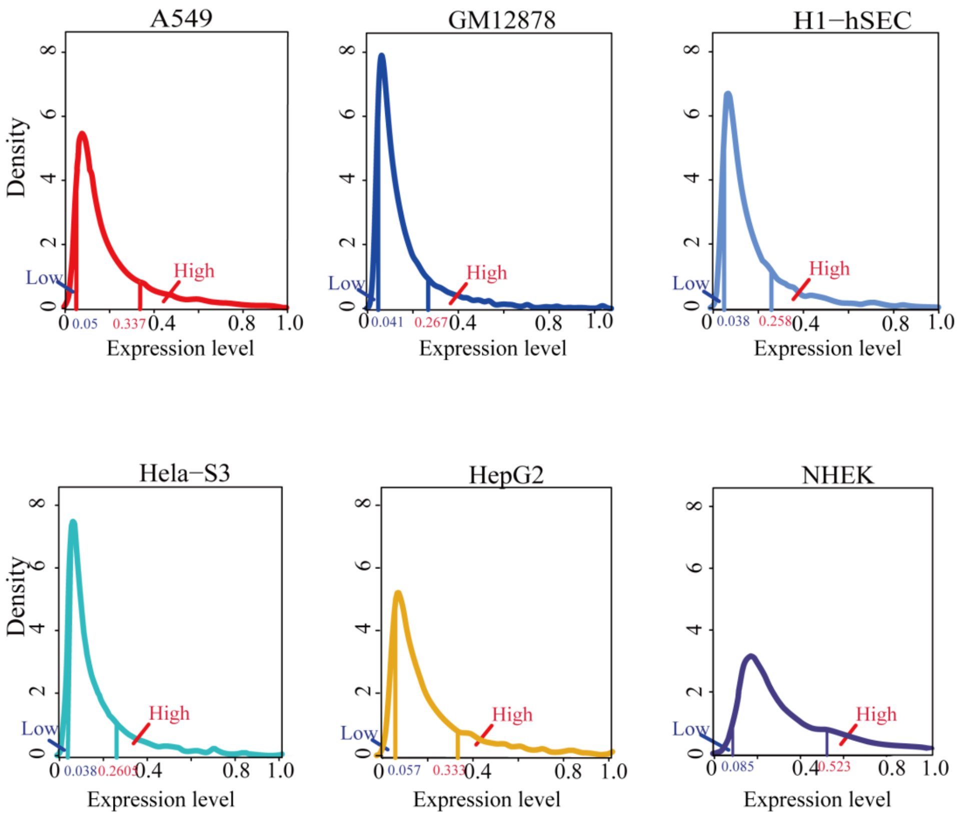

Figure S4. Density plots showing expression values distribution in six cell lines. A density map of expression values in six cell lines and a division of expression levels of high and low expression in each cell line. Related to Figure 2.

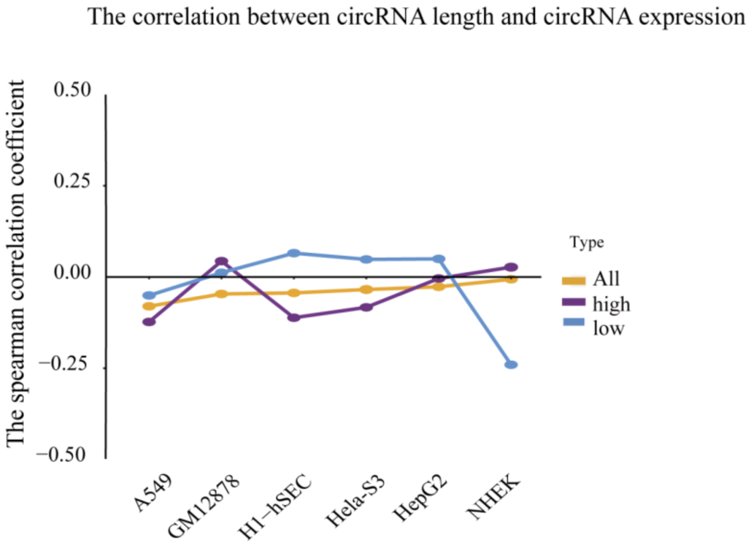

Figure S5. The correlation between circRNA length and circRNA expression. The spearman correlation of circRNA expression and circRNA length in all circRNAs, high expression of circRNA, low expression of circRNA in 6 cell lines. Related to Figure 2.

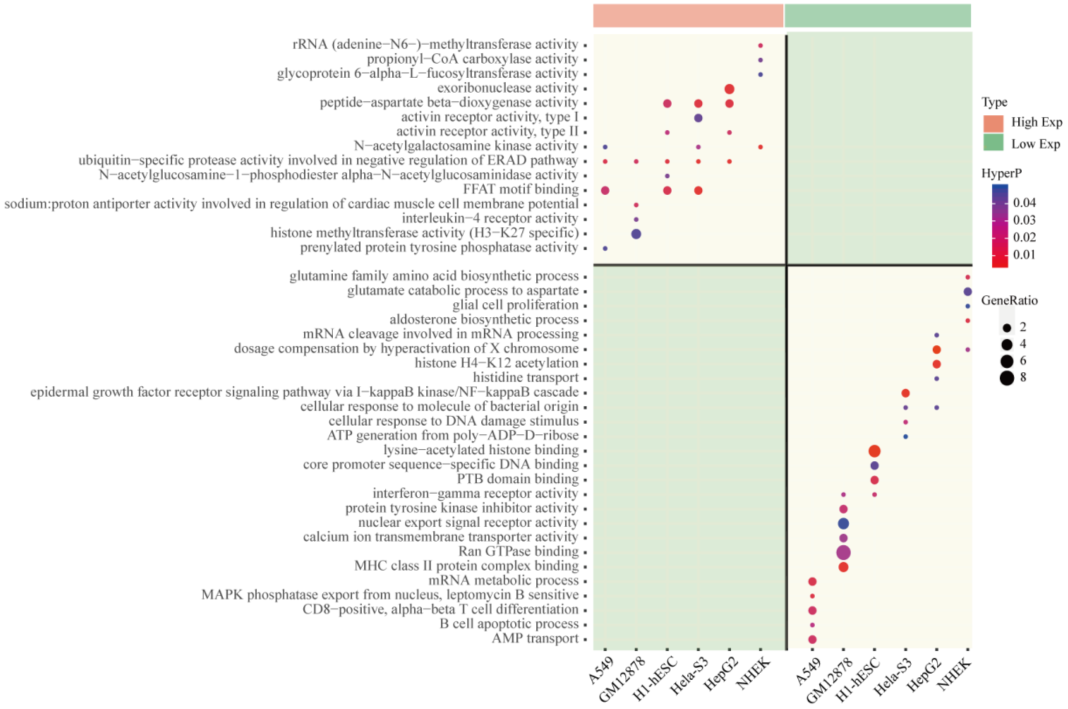

Figure S6. Enrichment and resistance events occur in circRNA high and low expression patterns. Enriched bubble diagrams of high and low expression patterns in molecular function. The portion covered by the red band represents a high expression circRNA, and the portion covered by the green band represents a low expression circRNA. The color of the bubble represents the p-value, bubble size represents the number of circRNA host genes which present in one term. Related to Figure 3.

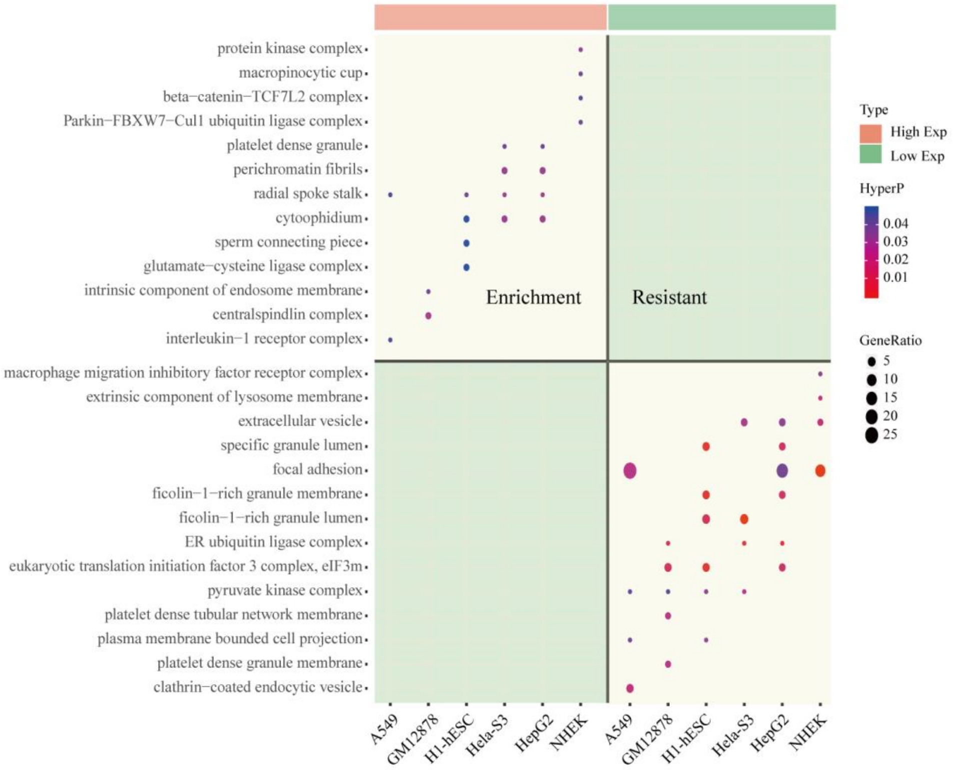

Figure S7. Enrichment and resistance events occur in circRNA high and low expression patterns. Enriched bubble diagrams of high and low expression patterns in cellular component. The portion covered by the red band represents a high expression circRNA, and the portion covered by the green band represents a low expression circRNA. The color of the bubble represents the p-value, bubble size represents the number of circRNA host genes which present in one term. Related to Figure 3.

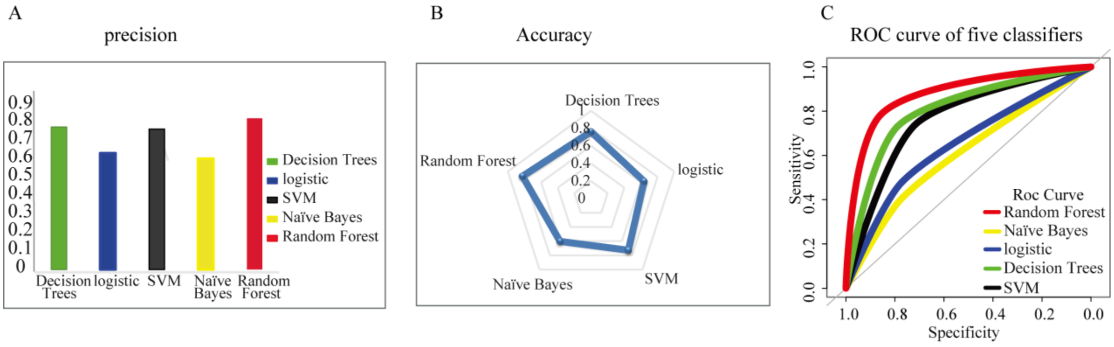

Figure S8. Evaluation of the effects of five classifiers in the A549 cell line. (A) Bar graph of the precision of the five classifiers. (B) Radar plots of the accuracy of the five classifiers. (C) The ROC curve of the five classifiers. Related to Figure 4.

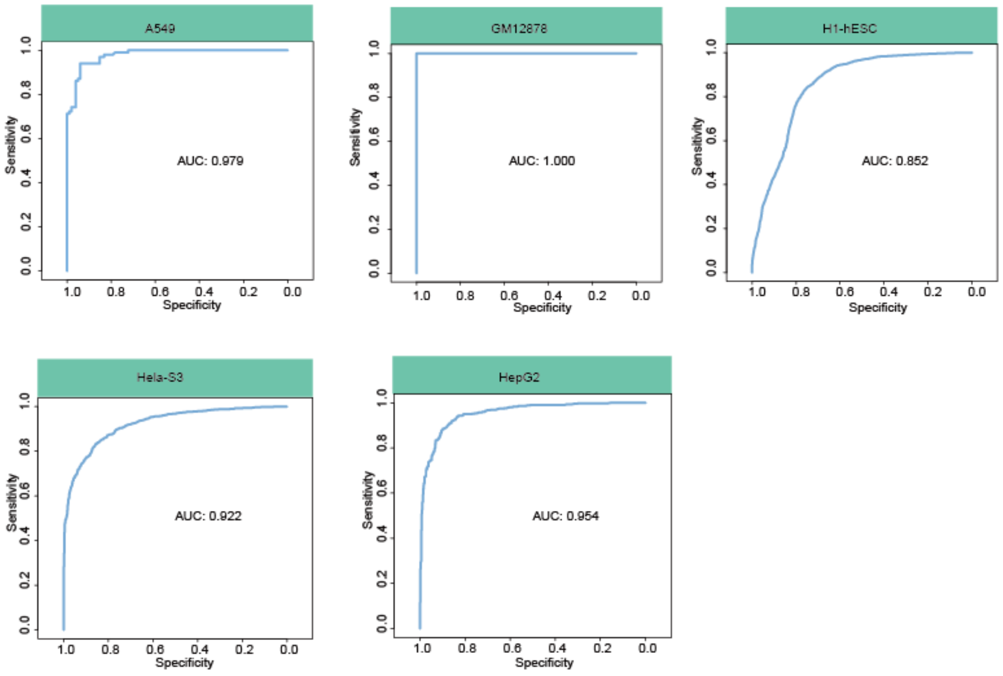

Figure S9. The area under the ROC curve of independent validation sets for each cell line. The predictive ability of top 5 factors to characterize the different expression patterns of circRNA. Related to Figure 5.

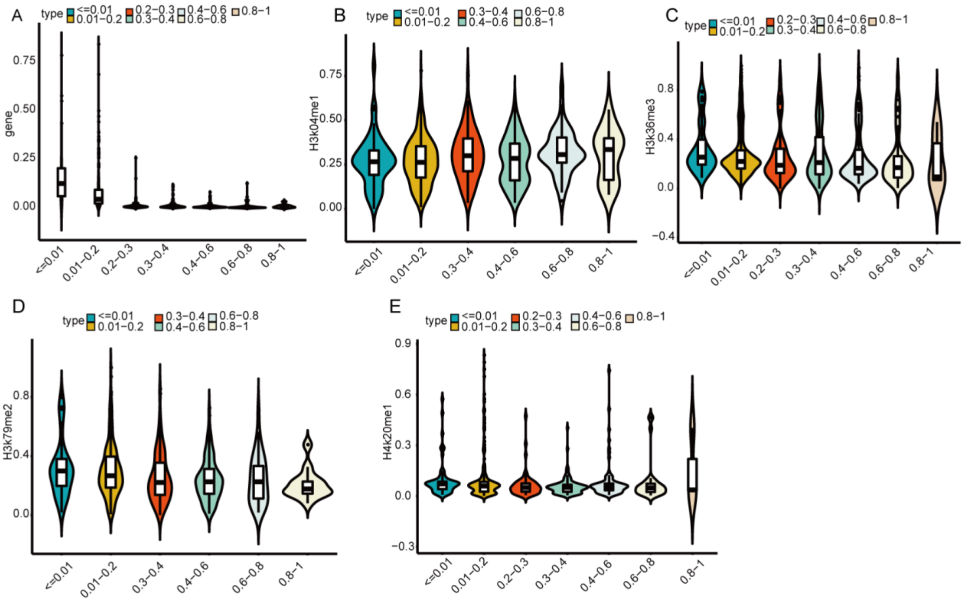

Figure S10. The relationship between circRNA expression and five important signals in the GM12878 cell line. (A) Violin map of host gene and circRNA expression. (B) Violin map of histone-modified H3K4me1 and circRNA expression. (C) Violin plot of histone modification of H3K36me3 and circRNA expression. (D) Violin plot of histone modification of H3K79me2 and circRNA expression. (E) Violin plot of histone-modified H4K20me1 and circRNA expression. Related to Figure 6.

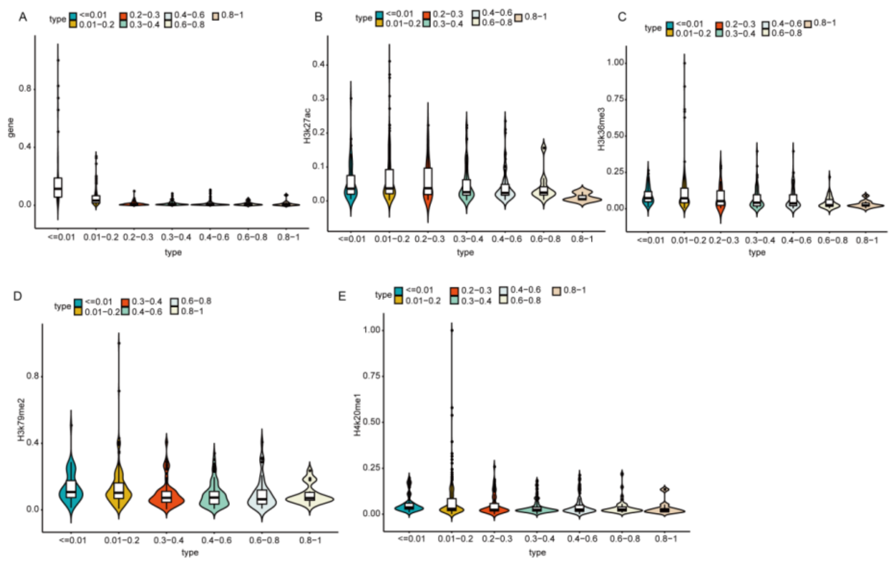

Figure S11. The relationship between circRNA expression and five important signals in the H1-hESC cell line. (A) Violin map of host gene and circRNA expression. (B) Violin map of histone-modified H3K27ac and circRNA expression. (C) Violin plot of histone modification of H3K36me3 and circRNA expression. (D) Violin plot of histone modification of H3K79me2 and circRNA expression. (E) Violin plot of histone-modified H4K20me1 and circRNA expression. Related to Figure 6.

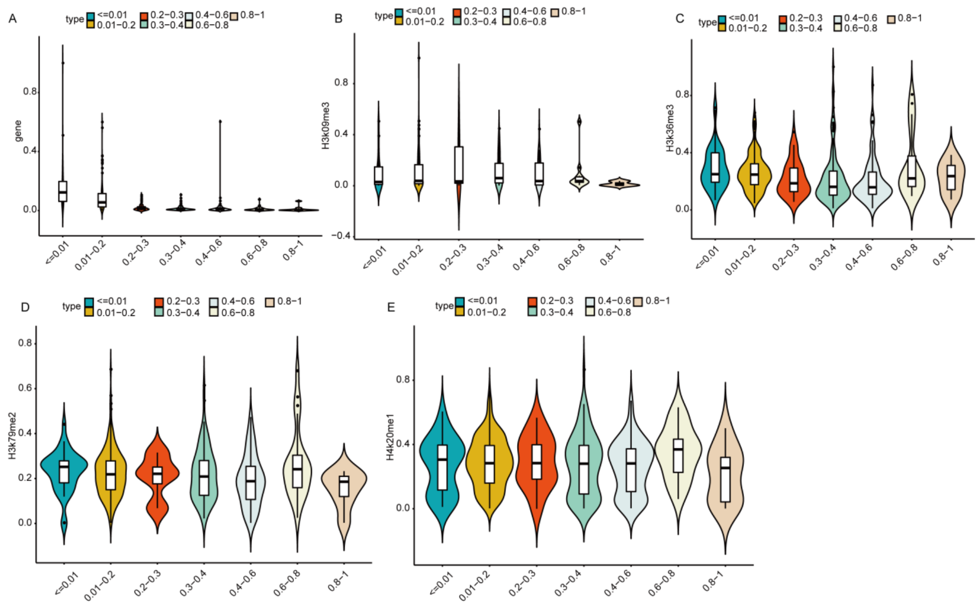

Figure S12. The relationship between circRNA expression and five important signals in the Hela-S3 cell line. (A) Violin map of host gene and circRNA expression. (B) Violin map of histone-modified H3K9me3 and circRNA expression. (C) Violin plot of histone modification of H3K36me3 and circRNA expression. (D) Violin plot of histone modification of H3K79me2 and circRNA expression. (E) Violin plot of histone-modified H4K20me1 and circRNA expression. Related to Figure 6.

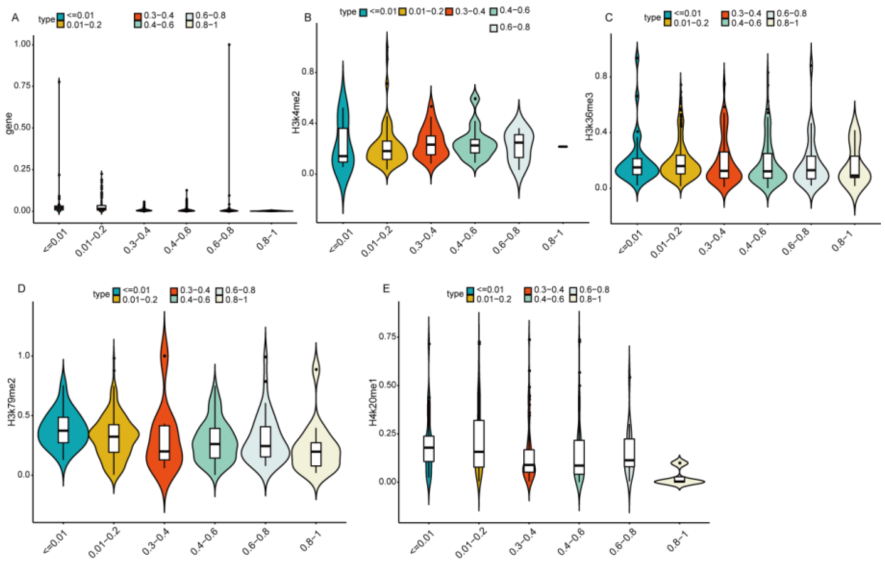

Figure S13. The relationship between circRNA expression and five important signals in the HepG2 cell line. (A) Violin map of host gene and circRNA expression. (B) Violin map of histone-modified H3K4me2 and circRNA expression. (C) Violin plot of histone modification of H3K36me3 and circRNA expression. (D) Violin plot of histone modification of H3K79me2 and circRNA expression. (E) Violin plot of histone-modified H4K20me1 and circRNA expression. Related to Figure 6.

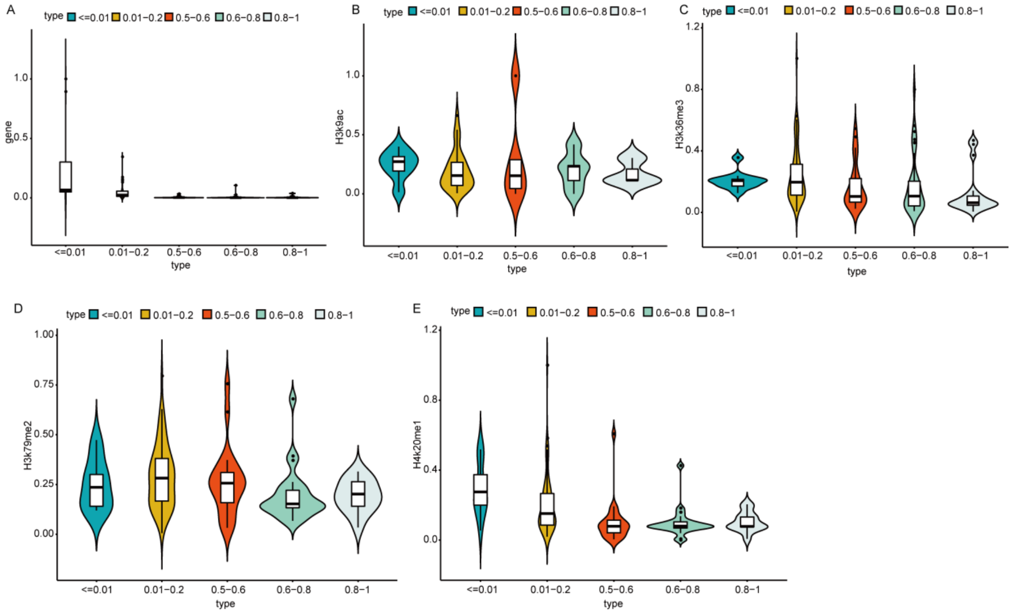

Figure S14. The relationship between circRNA expression and five important signals in the NHEK cell line. (A) Violin map of host gene and circRNA expression. (B) Violin map of histone-modified H3K9ac and circRNA expression. (C) Violin plot of histone modification of H3K36me3 and circRNA expression. (D) Violin plot of histone modification of H3K79me2 and circRNA expression. (E) Violin plot of histone-modified H4K20me1 and circRNA expression. Related to Figure 6.

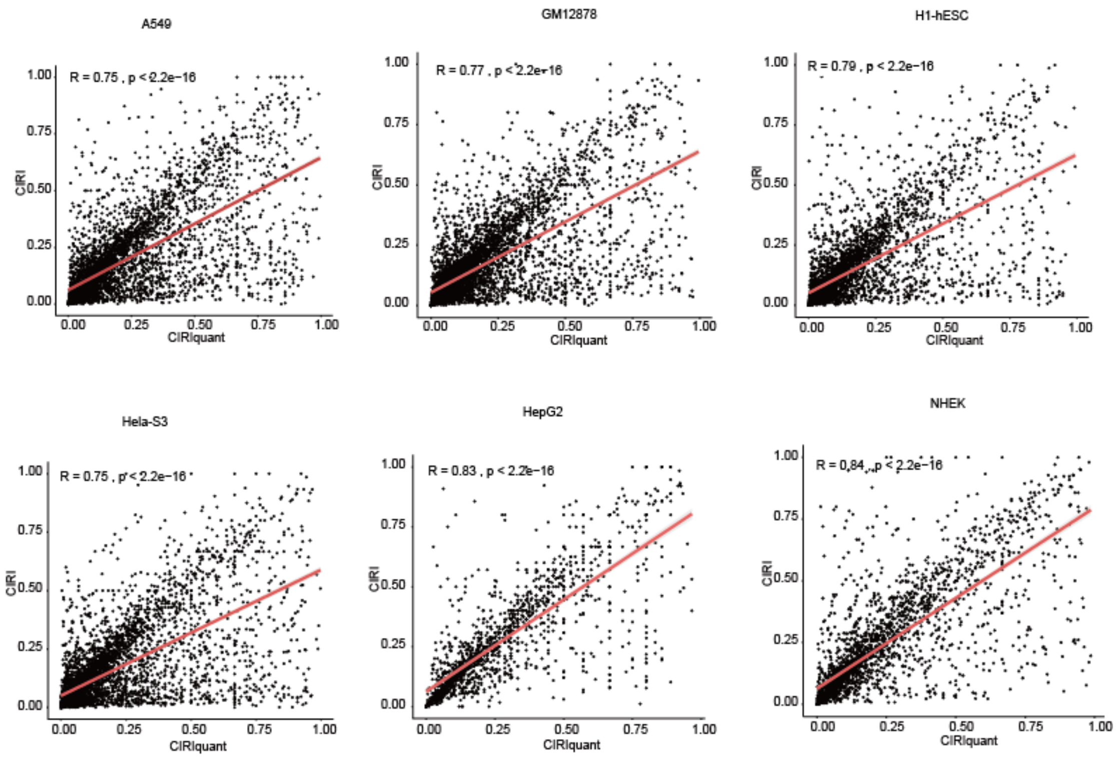

Figure S15. Scatter plot showing the correlation between the junction ratio in CIRI and CIRIquant, related to Figure 6.

Table S1. List of raw RNA-seq sequencing data for each cell line, related to Figure 1.

Cell line	Data
A549	SRR5048109.sra
	SRR5048110.sra
GM12878	SRR5048071.sra
	SRR5048072.sra
	SRR5048111.sra
	SRR5048112.sra
	SRR5048115.sra
	SRR5048116.sra
	SRR5048187.sra
Hela-s3	SRR5048117.sra
	SRR5048118.sra
	SRR5048119.sra
	SRR5048120.sra
	SRR5048132.sra
HepG2	SRR5048083.sra
	SRR5048084.sra
	SRR5048121.sra
	SRR5048122.sra
	SRR5048135.sra
	SRR5048136.sra
	SRR3192612.sra
NHEK	SRR3192482.sra
	SRR3192483.sra
	SRR3192484.sra
	SRR3192504.sra
	SRR3192505.sra
	SRR3192506.sra
H1-hESC	SRR5048069.sra
	SRR5048070.sra
	SRR5048129.sra
	SRR5048130.sra

Table S2. List of raw RNA-seq sequencing data for independent validation, related to Figure 4.

Cell line	Data
**A549**	SRR8371688.sra
	SRR8371689.sra
	SRR8371690.sra
**GM12878**	SRR3103887.sra
**H1-hESC**	SRR7230396.sra
	SRR7230403.sra
	SRR7230410.sra
**Hela-s3**	SRR8449597.sra
	SRR8449598.sra
	SRR8449599.sra
	SRR8449600.sra
**HepG2**	SRR4422330.sra
	SRR4422331.sra

Transparent Methods

RNA-Seq data in various cell lines. Raw sequencing data of RNA-Seq for six cell lines (including A549, GM12878, H1-hESC, HepG2, HeLa-S3 and NHEK) were downloaded from The Encyclopedia of DNA Elements (ENCODE) project (Consortium, 2004; Davis et al., 2018). Detailed samples information for cell lines was listed in Table S1. We next used fastQC (http://www.bioinformatics.babraham.ac.uk/projects/fastqc/) to evaluate the quality of the raw reads. Trimmomatic (Bolger et al., 2014) was used to filter low quality bases and adaptor sequences at both ends of the reads. Finally, clean reads were aligned to human reference genome (hg19) by TopHat v2.1.1 (Trapnell et al., 2009).

Identification of circRNAs and protein-coding genes in various cell lines. We applied CIRI (Gao et al., 2015) which is an efficiency and accuracy method to detect

the back-splice junction sites of circRNAs. In brief, we used FastQC for quality control of RNA-Seq data. BWA-MEM software (Li, 2013) was used to map reads to the reference genome hg19. We next used CIRI script to identify circRNAs in six cell lines. The default parameters were used for identifying circRNAs. The identified circRNAs were overlapped with circRNAs in circBase (Glazar et al., 2014), circAtlas (Wu et al., 2020) and circRIC (Ruan et al., 2019). The expressions of circRNAs were evaluated by relative counts of backsplice-junction reads and non-junction reads. We analyzed the genomic features of circRNAs, including circRNA types, circRNA exon length, and chromosome distribution. In addition, the expressions of protein-coding genes were calculated as Fragments Per Kilobase of exon per Megabase of library size (FPKM) by cufflinks v2.2.1 in each cell line (Trapnell et al., 2010).

Epigenetic modification of circRNAs and protein-coding genes. We utilized the co-localization approach to match histone modification signal of the circRNAs. In brief, 11 histone modification signals were downloaded in the University of California Santa Cruz (UCSC) Genome Browser database(W. James Kent et al., 2002) and matched to circRNAs according to the chromosomal location. If multiple histone modification peaks were aligned to the same circRNA, we used mean value as the circRNA-related histone modification signal. Moreover, circRNA-associated host gene expression levels were also defined by co-localization approach. For replicates of RNA-seq, we selected circRNAs that were shared in replicates for each cell line, and circRNA expression value was calculated as the mean value of all replicates.

Association analysis of circRNA expression. We used CIRIquant (Zhang et al., 2020)

to quantify the expression of circRNA with the following parameter settings: CIRIquant -t 4 -1 ./test_1.fq.gz -2 ./test_2.fq.gz --config ./chr.yml --no-gene- o ./test -p test. Next, the spearman correlation between the junction ratio in CIRI and the junction ratio in CIRIquant was calculated.

Construction of classifiers. We first analyzed the distribution of circRNA expression in six cell lines. We found that the majority of circRNAs were low level expression and the distribution exhibited a skewed distribution. The normalized expressions of circRNAs were around 0.1. To better distinguish the difference of high and low expressed circRNAs, we removed the circRNAs with moderate expression levels. Thus, we first ranked the circRNAs based on their expression levels and defined the top 20% and bottom 20% circRNAs as high and low expressed groups. CircRNA-related host genes and 11 histone modifications were selected to characterize circRNAs with different expression patterns. Next, 12 features were normalized to eliminate the dimension effect. The normalization formula is as follows:

$$z_i = \frac{x_i}{\sqrt{\sum_{i=0}^{n} x_i^2}} * 100 \quad (\text{Eq. } 1)$$

Finally, five classifiers including decision tree, logistic regression, SVM, naïve bayes, and random forest was constructed to predict circRNA expression patterns with the host gene and histone modifications as features.

Cross-validation of classifiers. We performed 10-fold cross validation for sampled datasets to evaluate the accuracy of the models. The process was repeated ten times

and the average area under the receiver operating characteristic curve (AUC) was calculated as the major indicator of prediction accuracy .For duplicate samples of each cell line, the test set in each cell line was set up to assess the robustness of the model according to the ratio n-1:1 (n represents the experimental number of replicates of the sample).

Independent validation set. The RNA-Seq datasets for independent validation were downloaded from the Gene Expression Omnibus database(GEO) (Ron Edgar et al., 2002). 11 histone modification signals were downloaded in The NIH Roadmap Epigenomics Mapping Consortium (http://www.roadmap epigenomics.org/). Detailed samples information for cell lines was listed in Table S2.

Function analysis of circRNAs. To explore the differences in the function of circRNA-related host genes with different expression classes, Gene Ontology (GO) terms and term gene sets in gene2Go were downloaded from National Center for Biotechnology Information (NCBI, https://www.ncbi.nlm.nih.gov/). KEGG pathways and pathway gene sets were obtained in The Molecular Signatures Database (Liberzon et al., 2011). Furthermore, we applied hyper-geometric distribution statistical theory to calculate p-values for enrichment analysis. We defined the over-represented and under-represented of host gene in the high and low expression patterns of circRNA across six cell lines. A function term presents over-represented when formula is satisfied and the p-value is under 0.05.

$$p(X > x) = \sum_{x}^{n} \frac{C_M^x C_{N-M}^{n-x}}{C_N^n} \text{(Eq. 2)}$$

A functional term was under-represented when formula is satisfied and the p-value is

under 0.05.

$$p(X \leq x) = 1 - \sum_{x}^{n} \frac{C_M^x C_{N-M}^{n-x}}{C_N^n} \text{(Eq. 3)}$$

Where x is the number of circRNA host genes annotated to a certain GO term, and N is the background gene sets. We selected the hg19 reference genome as the background gene sets. M is the number of all genes in a certain functional term, and n is the number of host genes of circRNAs.

Genome visualization of expression and histone modification. To discover the differences in modification signal of the important factors in circRNA, we further examined A549 cell line that is related to lung cancer. We obtained BigWig files of polyA RNA-seq, ployA deplept RNA-seq and histone modification peaks in A549 cell line from ENCODE (http://genome.ucsc.edu/ENCODE/downloads.html). The tool bigWigToBedGraph, which was acquired from UCSC, was used to convert BW files to BedGraph format. The WashU Epigenome Browser (https://epigenomegateway.wustl.edu/) was used to visualize high- and low-expression circRNA-modified signals.

Supplemental References：

Bolger, A.M., Lohse, M., and Usadel, B. (2014). Trimmomatic: a flexible trimmer for Illumina sequence data. Bioinformatics *30*, 2114-2120.

Consortium, E.P. (2004). The ENCODE (ENCyclopedia Of DNA Elements) Project. Science *306*, 636-640.

Davis, C.A., Hitz, B.C., Sloan, C.A., Chan, E.T., Davidson, J.M., Gabdank, I., Hilton, J.A., Jain, K., Baymuradov, U.K., Narayanan, A.K., *et al.* (2018). The Encyclopedia of DNA elements (ENCODE): data portal update. Nucleic acids research *46*, D794-D801.

Gao, Y., Wang, J., and Zhao, F. (2015). CIRI: an efficient and unbiased algorithm for de novo circular RNA identification. Genome biology *16*, 4.

Glazar, P., Papavasileiou, P., and Rajewsky, N. (2014). circBase: a database for circular RNAs. Rna *20*, 1666-1670.

Li, H. (2013). Aligning sequence reads, clone sequences and assembly contigs with BWA-MEM. arXiv *00*, 1–3.

Liberzon, A., Subramanian, A., Pinchback, R., Thorvaldsdottir, H., Tamayo, P., and Mesirov, J.P. (2011). Molecular signatures database (MSigDB) 3.0. Bioinformatics *27*, 1739-1740.

Ron Edgar, Michael Domrachev, and Lash, A.E. (2002). Gene Expression Omnibus: NCBI gene expression and hybridization array data repository. Nucleic acids research *30*, 207–210.

Ruan, H., Xiang, Y., Ko, J., Li, S., Jing, Y., Zhu, X., Ye, Y., Zhang, Z., Mills, T., Feng, J.*, et al.* (2019). Comprehensive characterization of circular RNAs in ~ 1000 human cancer cell lines. Genome Med *11*, 55.

Trapnell, C., Pachter, L., and Salzberg, S.L. (2009). TopHat: discovering splice junctions with RNA-Seq. Bioinformatics *25*, 1105-1111.

Trapnell, C., Williams, B.A., Pertea, G., Mortazavi, A., Kwan, G., van Baren, M.J., Salzberg, S.L., Wold, B.J., and Pachter, L. (2010). Transcript assembly and quantification by RNA-Seq reveals unannotated transcripts and isoform switching during cell differentiation. Nature biotechnology *28*, 511-515.

W. James Kent, Charles W. Sugnet, Terrence S. Furey, Krishna M. Roskin, Tom H. Pringle, Alan M. Zahler, and Haussler, D. (2002). The human genome browser at UCSC Genome research *12*, 996–1006.

Wu, W., Ji, P., and Zhao, F. (2020). CircAtlas: an integrated resource of one million highly accurate circular RNAs from 1070 vertebrate transcriptomes. Genome biology *21*.

Zhang, J., Chen, S., Yang, J., and Zhao, F. (2020). Accurate quantification of circular RNAs identifies extensive circular isoform switching events. Nature communications *11*, 90.
